# Serine 937 phosphorylation enhances KCC2 activity and strengthens synaptic inhibition

**DOI:** 10.1038/s41598-023-48884-x

**Published:** 2023-12-08

**Authors:** Tamara Radulovic, Ezhilarasan Rajaram, Lena Ebbers, Sara Pagella, Michael Winklhofer, Conny Kopp-Scheinpflug, Hans Gerd Nothwang, Ivan Milenkovic, Anna-Maria Hartmann

**Affiliations:** 1 Division of Physiology School of Medicine and Health Sciences, Carl Von Ossietzky University Oldenburg, 26111 Oldenburg, Germany; 2 Division of Neurobiology, Faculty of Biology, Ludwig-Maximilians-University Munich, 82152 Planegg-Martinsried, Germany; 3 Division of Neurogenetics, School of Medicine and Health Sciences, Carl Von Ossietzky University Oldenburg, 26111 Oldenburg, Germany; 4 Research Center Neurosensory Science, Carl Von Ossietzky University Oldenburg, 26111 Oldenburg, Germany; 5 Institute for Biology and Environmental Sciences IBU, Carl Von Ossietzky University of Oldenburg, 26111 Oldenburg, Germany; 6 Center of Excellence Hearing4all, Carl Von Ossietzky University Oldenburg, 26111 Oldenburg, Germany

**Keywords:** Auditory system, Cellular neuroscience, Development of the nervous system, Genetics of the nervous system, Neuronal physiology, Transporters in the nervous system

## Abstract

The potassium chloride cotransporter KCC2 is crucial for Cl^-^ extrusion from mature neurons and thus key to hyperpolarizing inhibition. Auditory brainstem circuits contain well-understood inhibitory projections and provide a potent model to study the regulation of synaptic inhibition. Two peculiarities of the auditory brainstem are (i) posttranslational activation of KCC2 during development and (ii) extremely negative reversal potentials in specific circuits. To investigate the role of the potent phospho-site serine 937 therein, we generated a KCC2 Thr^934Ala^/Ser^937Asp^ double mutation, in which Ser^937^ is replaced by aspartate mimicking the phosphorylated state, and the neighbouring Thr^934^ arrested in the dephosphorylated state. This double mutant showed a twofold increased transport activity in HEK293 cells, raising the hypothesis that auditory brainstem neurons show lower [Cl^−^]_i_. and increased glycinergic inhibition. This was tested in a mouse model carrying the same KCC2 Thr^934Ala^/Ser^937Asp^ mutation by the use of the CRISPR/Cas9 technology. Homozygous KCC2 Thr^934Ala^/Ser^937Asp^ mice showed an earlier developmental onset of hyperpolarisation in the auditory brainstem. Mature neurons displayed stronger glycinergic inhibition due to hyperpolarized E_Cl−_. These data demonstrate that phospho-regulation of KCC2 Ser^937^ is a potent way to interfere with the excitation-inhibition balance in neural circuits.

## Introduction

Proper brain function relies on a fine tuned balance between excitation and inhibition^1,2^. Excitatory synaptic transmission is mainly mediated through glutamatergic synapses and inhibitory synaptic transmission through GABAergic and glycinergic signalling^2^. Binding of GABA (gamma aminobutyric acid) and glycine to ionotropic GABA_A_ and glycine receptors, respectively^3^, results in Cl^-^ influx into mature neurons leading to hyperpolarizing inhibitory postsynaptic potentials (IPSPs). In contrast, in immature neurons GABA and glycine cause a depolarisation of the membrane due to Cl^-^ efflux driven by high [Cl^-^]_i_^4–9^. Depolarisation in immature neurons facilitates opening of voltage-gated Ca^2+^ channels thereby evoking local Ca^2+^ transients^10–16^. Ca^2+^ in turn activates intracellular signalling cascades, which are important to establish and stabilize synaptic connections^12,15,17^. The developmental shift from depolarisation to hyperpolarisation (D/H shift) is conserved throughout the nervous system (e.g. brainstem, cortex, hippocampus, hypothalamus, and spinal cord)^4,6,8,12,13,18–20^ and occurs during early postnatal life in most rodents^21,22^.

The key players regulating the D/H shift are NKCC1 (sodium potassium chloride cotransporter 1) and KCC2 (potassium chloride cotransporter 2)^23–26^. Both membrane transporters are secondary active mediating the Cl^-^ coupled transport of K^+^ and/or Na^+^^27,28^. In immature neurons, NKCC1 is the predominant transporter mediating Cl^-^ uptake and intracellular accumulation^29–32^. In mature neurons, KCC2 is the central Cl^-^ extruder that lowers [Cl^−^]_i_ and thus enables fast hyperpolarizing postsynaptic inhibition^33,34^. The physiological relevance of NKCC1 and KCC2 is underlined by the severe phenotypes of knock-out mice models. Mice with disruption of the gene *Slc12a5* encoding both splice variants of KCC2 (KCC2a and KCC2b) die shortly after birth due to severe motor deficits that compromise respiration^35,36^. Mice with disruption of the gene *Slc12a2* encoding NKCC1 are viable, but suffer from deafness, increased pain perception, and male infertility^31,37–39^.

In most brain areas including cortex, hippocampus and the cerebellum, the D/H shift is caused by a decrease in NKCC1 and an increase in KCC2 expression during development^31,33,40–46^. Interestingly, auditory brainstem neurons perinatally express KCC2 at high levels in the plasma membrane, but in a transport-inactive form^47–49^. Here, the developmental increase in KCC2 transport activity correlates with an increase in the oligomer/monomer ratio^49^ and a switch from membrane rafts to non-membrane rafts^50^. In addition, phospho-regulation may play an essential role in the developmental activation of KCC2^51,52^, but detailed knowledge of the multiple phosphorylation sites is lacking.

In the mature auditory brainstem, synaptic inhibition is key in various computational tasks during neural signal processing. This is reflected by well-defined inhibitory projections and circuit-specific hyperpolarizing glycine reversal potentials (E_Gly_). The superior paraolivary nucleus (SPN) is involved in encoding sound offsets^53^ and displays an exceptionally negative E_Gly_ =  − 90.6 mV, compared to other auditory nuclei, such as the auditory lateral superior olive (LSO) (E_Gly_ =  − 68.6 mV) and the medial superior olive (MSO) (E_Gly_ =  − 75.8 mV) in mice^54^.

**Figure 1:**
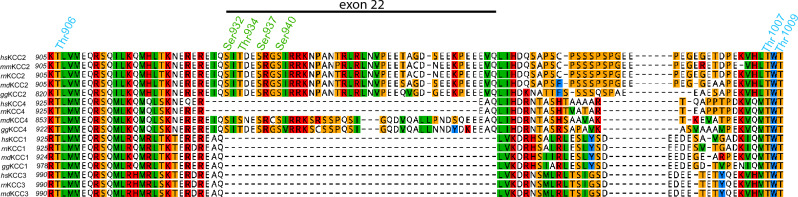
Evolutionary conservation of phosphorylation site in vertebrate KCC isoforms. Multialignment of a C-terminal part of vertebrate KCCs were generated using ClustalW in Genious. Amino acid residues encoded by exon 22 are marked by a black bar. Exon 22 is only present in all vertebrate KCC2 and non-therian KCC4 isoforms. Phosphorylation sites that enhance KCC2 activity upon dephosphorylation are marked with blue asterisks. These phosphorylation sites are Thr^906^, Thr^1007^ and Thr^1009^. They are highly conserved among all vertebrate KCC isoforms. Phosphorylation sites that are marked with green asterisks enhance KCC2 activity upon phosphorylation. These sites are Ser^932^, Thr^934^, Ser^937^, and Ser^940^. They are only present in KCC isoforms encoded by exon 22. *hs* (*Homo sapiens*), *mm* (*mus musculus), rn* (*Rattus norvegicus*), *md* (*Monodelphis domesticus*), *gg* (*Gallus gallus*).

Differential phosphorylation of KCC2 could be one mechanism underlying different E_Gly_ in neuronal subtypes. KCC2 harbours several phosphorylation sites that are present at the N-terminus and C-terminus and have been shown to impact its transport activity^28,55–57^. Interestingly, both dephosphorylation of Thr^6^ in KCC2a, Thr^906^, Thr^1007^, Thr^1009^, and Tyr^1087^ and phosphorylation of Ser^932^, Thr^934^, Ser^937^, and Ser^940^ enhances KCC2 activity^52,57–69^. The latter phospho-sites are all encoded by exon 22, which is only present in KCC2 and non-therian KCC4 (Fig. 1)^65,66,69–71^. These complex phosphorylation and dephosphorylation combinations provide KCC2 with a rich-regulatory tool-box to achieve graded activity and the integration of different signalling pathways^60,66^.

Here, we used CRISPR/Cas9 gene editing to generate transgenic mice with a Thr^934Ala^/Ser^937Asp^ double mutation in KCC2 to probe for a functional link between KCC2 phosphorylation and its transport activity.

## Results

### Phosphorylation of Ser^937^ in the KCC2a Thr^934Ala^/Ser^937Asp^ double mutant enhances KCC2 transport activity

**Figure 2:**
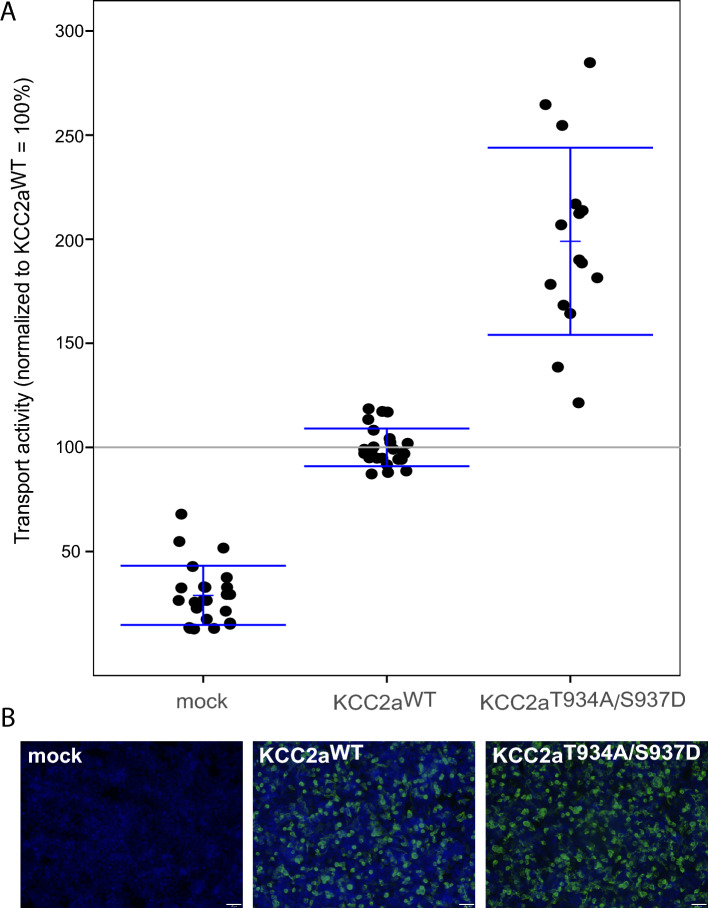
The KCC2 Thr^934Ala^/Ser^937Asp^ double mutant enhances KCC2 activity. HEK293 cells were transiently transfected with KCC2a^WT^ or KCC2a Thr^934Ala^/Ser^937Asp^. (**A**) Tl^+^ flux measurement was performed to determine the KCC2 transport activity. KCC2a^WT^ displayed a significant higher transport activity (100 ± 1.8%, *p* = 1.93 × 10^−10^) than mock transfected cells (24 ± 2.9%). The KCC2a Thr^934Ala^/Ser^937Asp^ double mutant (199 ± 11.6%) significantly enhances KCC2 transport activity (*p* = 0.001). The graph represents the data of at least five independent measurements including three technical replicates per independent measurement, normalized to KCC2a^WT^. Depicted are mean values ± SE. (**B**) Immunocytochemistry was used to monitor the transfection rate of KCC2 (green) and cell staining by DAPI (blue). The scale bar is 200 µm.

Serine 937 represents an experimentally proven phosphorylation site^58,65^. It might sometimes be flanked by phosphorylation of threonine 934^58^. A previous analysis demonstrated that single mutation of threonine 934 or serine 937 into aspartate (mimicking the phosphorylated state) increases KCC2 activity twofold, whereas mutation of either of the two residues into alanine (mimicking the dephosphorylated state) does not impair KCC2 activity^65^. Here, we generated a KCC2 Thr^934Ala^/Ser^937Asp^ double mutant that traps Ser^937^ in the phosphorylated state (Thr^934Ala^) and the nearby Thr^934^ in the dephosphorylated state (Ser^937Asp^). Importantly, the latter mutation Thr^934Ala^ alone does not alter KCC2 activity^65^. This Thr^934Ala^/Ser^937Asp^ mutant enabled us to specifically investigate the effect of Ser^937^ phosphorylation on the activity. The double mutation also prevents a possible confounding action of phosphatases and kinases on threonine 934, which might render interpretation of the results difficult. Immunocytochemical analyses of the transiently transfected KCC2 constructs in HEK293 cells revealed similar transfection rates between KCC2a wild-type (KCC2a^WT^) and KCC2a Thr^934Ala^/Ser^937Asp^ (Fig. 2B). To analyse the transport activity, we used the well-established Tl^+^ flux measurement method^65,66,72,73^. HEK293 cells transfected with KCC2a^WT^ showed higher transport activity than in mock transfected cells (100 ± 9.05% vs. 28.92 ± 14.23%; *p* = 1.93 × 10^−10^) (Fig. 2A, Table 1). In comparison, the KCC2a Thr^934Ala^/Ser^937Asp^ double mutant showed twofold higher transport activity compared to KCC2a^WT^ (199 ± 44.96%; *p* = 0.001) (Fig. 2A, Table 1). These data reveal that phosphorylation of Ser^937^ in Thr^934Ala^/Ser^937Asp^ double mutant enhances the activity.

**Table 1:** Transport activity of KCC2a constructs.

	Average and SE	Significance in comparison to KCC2^wt^	Significance in comparison to mock
mock	24% ± 2.9%	***	–
KCC2^wt^	100% ± 1.8%	–	***
Thr^934Ala^/Ser^937Asp^	199% ± 11.6%	***	***

***p < 0.001.

### Homozygous KCC2 Thr^934Ala^/Ser^937Asp^ mice show no obvious phenotypic changes

**Figure 3:**
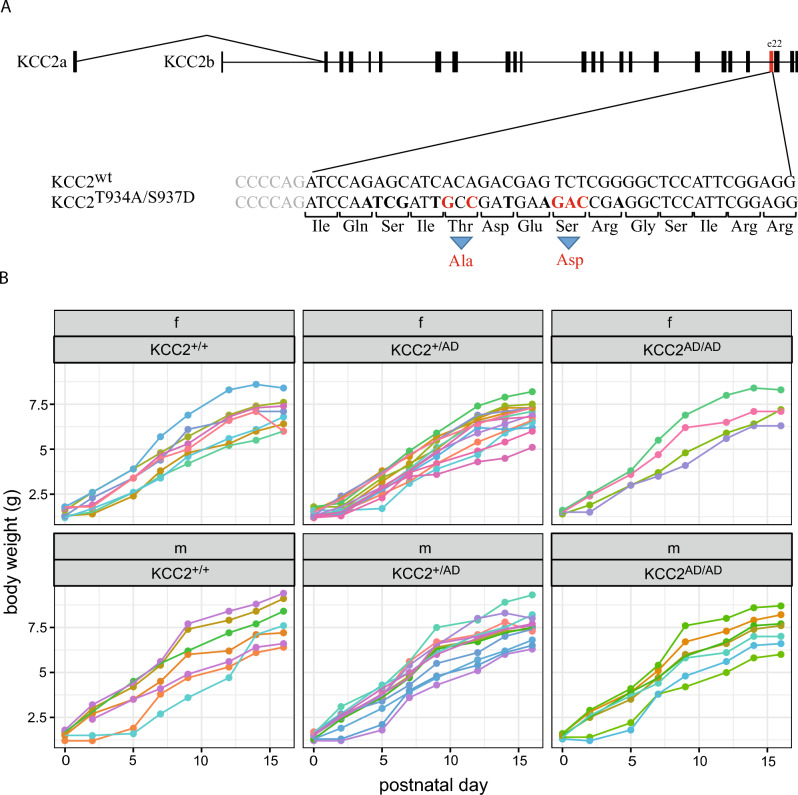
Characterization of transgenic KCC2 Thr^934Ala^/Ser^937Asp^ mice. (**A**) The transgenic KCC2 Thr^934Ala^/Ser^937Asp^ mouse line was generated with CRISPR/Cas. Here, Thr^934^ was mutated to alanine and Ser^937^ was mutated to aspartate. Both phosphorylation sites are located in exon 22. (**B**) Compared to KCC2^+/+^ littermates (n = 15), heterozygous (KCC2^+/AD^, n = 26) and homozygous (KCC2^AD/AD^, n = 12) mice show no differences in weight gain from P0 to P16 (Tables 3 and 4).

To analyse the in vivo impact of Ser^937^ phosphorylation, we generated a KCC2 Thr^934Ala^/Ser^937Asp^ mice via CRISPR/Cas9 located in exon 22 (Fig. 3A). The two mutations were verified by genotyping and DNA sequencing. Heterozygous and homozygous KCC2 Thr^934Ala^/Ser^937Asp^ mice, which we here name KCC2^+/AD^ (heterozygous) and KCC2^AD/AD^ (homozygous) are viable, fertile, showing no abnormalities in the nutritional state, posture, motor skills and coat. The genotypes of the littermates show the expected Mendelian inheritance of the two alleles (χ^2^-test 1:2:1; Table 2). Compared to KCC2^+/+^ littermates, heterozygous KCC2^+/AD^ and homozygous KCC2^AD/AD^ mice show no differences in weight gain from P0 to P16 (Fig. 3B, Table 3, *p*-value = 0.76, see ANOVA output in Table 4). As usual, male mice become slightly heavier than females (*p*-value = 0.025, see Table 4), without a genotype-specific effect (*p*-value = 0.65). Thus, heterozygous KCC2^+/AD^ and homozygous KCC2^AD/AD^ mice show no obvious phenotypic changes.

**Table 2:** Chi^2^ test of the birth rate according to Mendel.

KCC2	Number of littermates		Chi^2^	*df*	*p*-value
	Observed	Expected			
+/+	15	13.25	0.36	2	0.83
+/AD	26	26.5			
AD/AD	12	13.25			

+/+: Homozygous KCC2^WT^ mice, +/AD: heterozygous Thr^934Ala^/Ser^937Asp^ mice, AD/AD: homozygous Thr^934Ala^/Ser^937Asp^ mice.

**Table 3:** Weight of KCC2 mice.

KCC2	Sex	n	P0	P2	P5	P7	P9	P12	P14	P16
+/+	Female	8	1.50 g ± 0.25 g	1.98 g ± 0.47 g	3.20 g ± 0.59 g	4.35 g ± 0.77 g	5.33 g ± 0.88 g	6.41 g ± 1.03 g	6.89 g ± 0.98 g	6.96 g ± 0.83 g
	Male	7	1.55 g ± 0.21 g	2.40 g ± 0.76 g	3.37 g ± 1.18 g	4.51 g ± 1.07 g	5.79 g ± 1.48 g	6.47 g ± 1.39 g	7.37 g ± 0.99 g	7.81 g ± 1.18 g
+/AD	Female	14	1.43 g ± 0.22 g	1.74 g ± 0.36 g	3.00 g ± 0.61 g	3.99 g ± 0.57 g	4.89 g ± 0.71 g	6.09 g ± 0.92 g	6.53 g ± 0.89 g	6.86 g ± 0.76 g
	Male	12	1.48 g ± 0.17 g	2.33 g ± 0.57 g	3.46 g ± 0.79 g	4.72 g ± 0.66 g	5.95 g ± 0.95 g	6.65 g ± 0.91 g	7.26 g ± 0.87 g	7.50 g ± 0.80 g
AD/AD	Female	5	1.48 g ± 0.08 g	2.10 g ± 0.41 g	3.32 g ± 0.36 g	4.30 g ± 0.81 g	5.48 g ± 1.11 g	6.34 g ± 0.99 g	6.96 g ± 0.86 g	7.12 g ± 0.75 g
	Male	7	1.47 g ± 0.11 g	2.27 g ± 0.68 g	3.30 g ± 0.91 g	4.56 g ± 0.61 g	5.86 g ± 1.13 g	6.47 g ± 1.01 g	7.26 g ± 0.92 g	7.40 g ± 0.93 g

+/+: Homozygous KCC2^WT^ mice, +/AD: heterozygous Thr^934Ala^/Ser^937Asp^ mice, AD/AD: homozygous Thr^934Ala^/Ser^937Asp^ mice.

**Table 4:** Two-way repeated measurement ANOVA of weight of KCC2 mice.

Factor	Df	Sum Sq	Mean Sq	*F* value	*p*-value
Sex	1	21.28	21.283	5.351	0.025
Genotype	2	2.19	1.094	0.275	0.76
Sex:genotype	2	3.40	1.701	0.428	0.65
(Residuals)	46	182.97	3.978		

Df: Degrees of freedom, Sum Sq: Sum squares, Mean Sq: mean squares, *F*: *F* (mean Sq(factor)/MeanSq (res), Df(factor), Df(res)).

**Figure 4:**
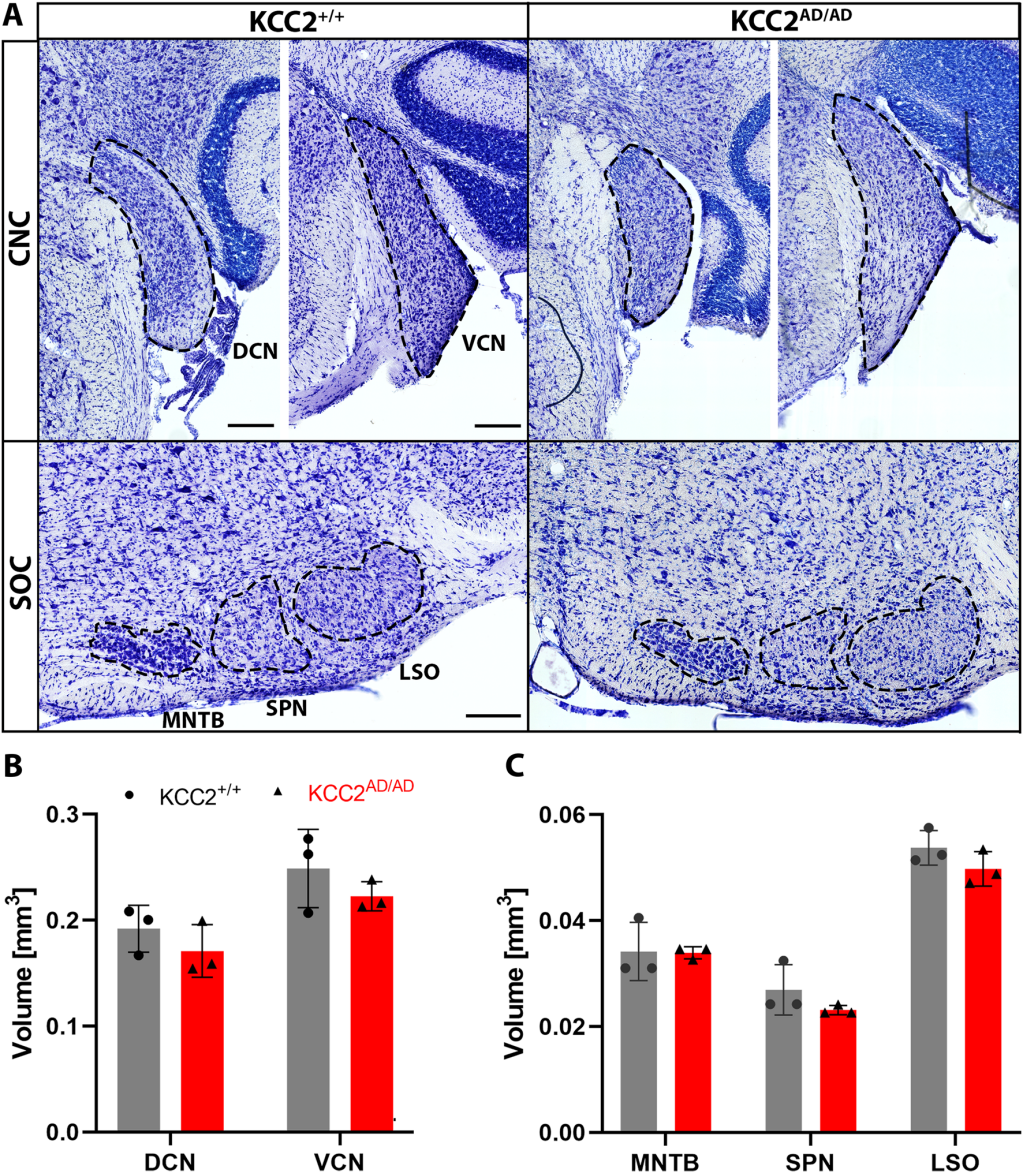
No morphological changes in the auditory brainstem of homozygous KCC2 Thr^934Ala^/Ser^937Asp^ mice. (**A**) Representative images of Nissl-stained sections of the auditory brainstem nuclei of adult homozygous KCC2^WT^ (KCC2^+/+^) and KCC2 Thr^934Ala^/Ser^937Asp^ (KCC2^AD/AD^) mice. Quantitative morphometric analyses revealed no differences in volume of the nuclei of the CNC (**B**) as well as of the SOC (**C**) between the two genotypes. Depicted are mean values ± SD, N = 3 mice/genotype; scale bar = 200 µm. CNC, cochlear nucleus complex; DCN, dorsal cochlear nucleus; LSO, lateral superior olive; MNTB, medial nucleus of the trapezoid body; SOC, superior olivary complex; SPN, superior paraolivary nucleus; VCN, ventral cochlear nucleus.

Next, we performed morphometric measurements in the auditory brainstem. Quantitative analysis of Nissl-stained sections of the brainstem revealed no significant changes in volume of major auditory nuclei. Both the dorsal (DCN) and ventral (VCN) parts of the cochlear nucleus complex (DCN: KCC2^+/+^: 0.192 ± 0.022 mm^3^, KCC2^AD/AD^: 0.171 ± 0.025 mm^3^, *p* = 0.334; VCN: KCC2^+/+^: 0.249 ± 0.037 mm^3^, KCC2^AD/AD^: 0.223 ± 0.014 mm^3^, *p* = 0.315; Fig. 4A,B) were not affected. Nuclei of the superior olivary complex showed also no morphological differences. All three examined nuclei, the lateral superior olive (LSO), the superior paraolivary nucleus (SPN), and the medial nucleus of the trapezoid body (MNTB) showed no change in volume (LSO: KCC2^+/+^: 0.054 ± 0.003 mm^3^, KCC2^AD/AD^: 0.050 ± 0.003 mm^3^, *p* = 0.208; SPN: KCC2^+/+^: 0.027 ± 0.005 mm^3^, KCC2^AD/AD^: 0.023 ± 0.001 mm^3^, *p* = 0.239; MNTB: KCC2^+/+^: 0.034 ± 0.005 mm^3^ KCC2^AD/AD^: 0.034 ± 0.001 mm^3^, *p* = 0.315; Fig. 4A,C). Thus, the phosphorylation mimic of Ser^937^ does not affect gross morphology of the auditory brainstem, probably ruling out effects on cell migration or cell viability.

### Earlier D/H shift in LSO neurons of homozygous KCC2 Thr^934Ala^/Ser^937Asp^ mice

**Figure 5:**
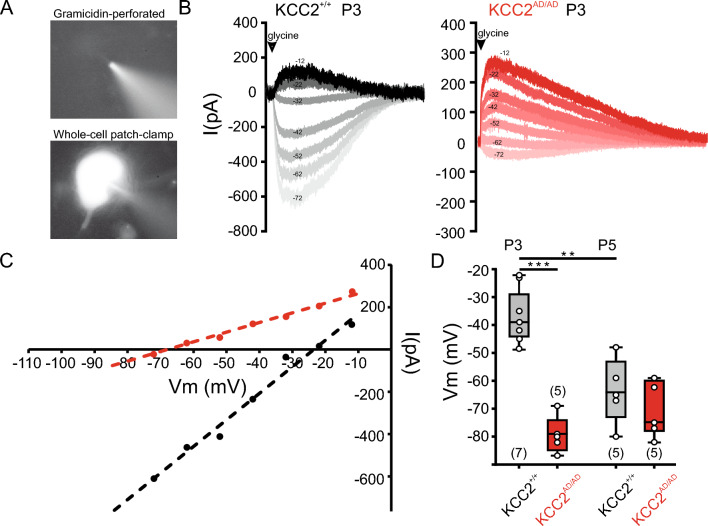
Earlier D/H shift in homozygote KCC2 Thr^934Ala^/Ser^937Asp^ mice. Gramicidin perforated recording in LSO neurons. (**A**) *(up)* image of gramicidin perforated recording, visible patch pipette loaded with Atto 488; *(below)* whole-cell recording configuration with Atto488 inside the cell following patch rupture. (**B**) Representative traces from recordings made in control (KCC2^+/+^) and homozygous KCC2 Thr^934Ala^/Ser^937Asp^ (KCC2^AD/AD^) group at P3 showing current traces in response to glycine application at different holding potentials. (**C**) Representative plot of peak current amplitudes at different voltages for both groups at P3 (black control, red KCC2^AD/AD^). Line intersection with x-axis indicates the E_Cl−_. (**D**) Population data from control and KCC2^AD/AD^ at P3 and P5 showing significantly hyperpolarized E_Cl−_ at P3 in KCC2^AD/AD^ compared to control. The control group shows a developmental change towards hyperpolarized values from P3 to P5.

The action of glycine, the main inhibitory neurotransmitter in the brainstem, shows a D/H shift between P4 and P8 in LSO neurons^8,20,47,74,75^. To access whether phosphorylation of KCC2 affects its transport activity, we performed gramicidin perforated patch clamp recordings in LSO neurons at the ages P3 and P5, as previously described^76–78^. To this end, the reversal potential of glycine-evoked currents was determined while leaving the [Cl^−^]_i_ undisturbed through gramicidin-pores that are exclusively permeable to monovalent cations and small, uncharged molecules^79,80^. Glycine was applied via puff-application (100 ms) at saturating concentration of 1 mM, while neurons were held at different membrane potentials. Synaptic stimulation can not be used to reliably estimate E_Cl−_ at this immature stage, due to ongoing development of inhibitory inputs^20,81–83^. In control mice, we measured a depolarized E_Cl−_ at P3 compared to P5 (Fig. 5) and hypothesized in homozygous KCC2^AD/AD^ mice a more hyperpolarized E_Cl−_ with respect to controls. In the control data set containing cells from wild type (KCC2^+/+^) littermates and C57Bl/6N animals, we recorded the average E_Cl−_ at P3 =  − 36.6 mV (n = 7) and at P5 =  − 63.8 mV (n = 5), in agreement with a developmental shift toward hyperpolarizing values (**, *p* = 0.002). In contrast, recordings in KCC2^AD/AD^ LSO neurons showed the average E_Cl−_  =  − 79.4 mV (n = 5) already at P3, with no further change at P5 (E_Cl−_  =  − 71 mV, n = 5, *p* = 0.155). These data demonstrate that the phospho-mimetic mutation of KCC2 at Ser^937^ causes an early D/H shift of E_Cl−_ compared to the control (− 79.4 mV vs. − 36.6 mV at P3, ***p < 0.001), suggesting an important role of this phospho-site in developmental regulation of KCC2.

### Adult homozygous KCC2 Thr^934Ala^/Ser^937Asp^ mice show more hyperpolarized IPSC-reversal potentials

**Figure 6:**
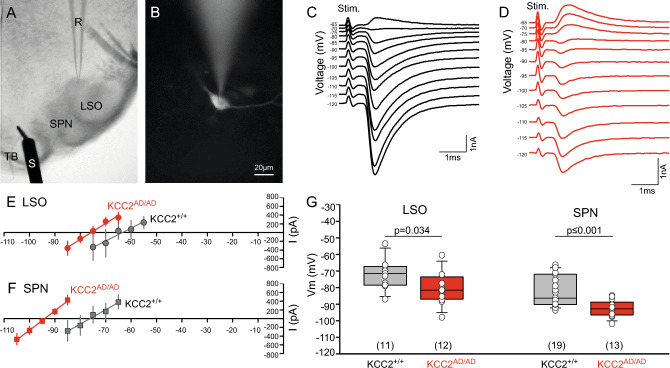
Hyperpolarized E_Cl−_ in mature LSO and SPN neurons of homozygous KCC2 Thr^934Ala^/Ser^937Asp^ mice. (**A**) Coronal slice at the level of the superior olive showing the bipolar stimulation electrode (left) in the MNTB and a patch pipette (right) in the LSO. (**B**) Low magnification image of an LSO neuron filled with dye via the patch pipette. (**C**), (**D**) Synaptically evoked IPSCs recorded in LSO neurons of wild type (KCC2^+/+^) (**C**) and homozygous KCC2 Thr^934Ala^/Ser^937Asp^ (KCC2^AD/AD^) mice (**D**) over a range of holding potentials (− 120 to − 65 mV). (**E**), (**F**) The mean I/V relationship for the IPSCs in wild type neurons (black) and KCC2^AD/AD^ neurons (red) revealed a left shift towards more hyperpolarized IPSC reversal potentials (= line intersection with x-axis) in the LSO (**E**) and the SPN (**F**). (**G**) This difference in reversal potentials was significant for mature neurons in the LSO and SPN.

Based on the increased Cl^−^ extrusion activity of KCC2 observed in HEK293 cells and immature neurons, we predicted more hyperpolarized reversal potentials for synaptically evoked inhibition (E_IPSCs_) in mature LSO and SPN neurons. We tested this hypothesis by taking advantage of whole cell patch-clamp recordings that allowed to set high intracellular Cl^-^ concentrations of 34.5 mM in order to further challenge KCC2’s Cl^-^ extrusion activity. Based on the Nernst equation, the compositions of pipette solution, the ACSF, the junction potential correction and the assumption that glycinergic synaptic currents are mainly carried by Cl^−^, the predicted E_IPSC_ was − 36 mV. However, electrical stimulation of the inhibitory fibre tracts leaving the MNTB, the major source of glycinergic inhibition in the superior olivary complex (Fig. 6A,B), reliably elicited pharmacologically isolated glycinergic IPSCs with E_IPSCs_ of at least − 70 mV in wild type KCC2^+/+^ and homozygous KCC2^AD/AD^ mice. The driving force of the IPSCs, or else the strength of inhibition, is governed by the difference between the resting (in our case holding) potential and the reversal potential of the IPSC. IPSCs of homozygous KCC2^AD/AD^ neurons reversed at more hyperpolarized membrane potentials compared to wild type neurons, suggesting a larger driving force for inhibition in homozygous KCC2^AD/AD^ mice and thus increased KCC2 activity (Fig. 6C,D). These more hyperpolarizing IPSCs resulted in a leftward shift of the current–voltage relationship in both LSO and SPN neurons (Fig. 6E,F), which changed from − 72.0 ± 2.6 mV (n = 11) in LSO wild type neurons to − 80.6 ± 2.7 mV (n = 12) KCC2^AD/AD^ neurons (two-tailed *t*-test: *p* = 0.034; Fig. 6G). A hyperpolarizing shift was also observed from − 82.0 ± 2.1 mV (n = 19) in wild type SPN neurons to − 92.8 ± 1.4 mV (n = 13) KCC2^AD/AD^ neurons (two-tailed *t*-test: *p* ≤ 0.001; Fig. 6G). Thus, the phospho-mimetic mutation at Ser^937^ affects not only the developmental shift in immature auditory neurons, but also increases the driving force for inhibition in mature neurons.

## Discussion

We generated a transgenic KCC2 Thr^934Ala^/Ser^937Asp^ mouse, in which serine 937 is replaced by aspartate mimicking the phosphorylated state and analysed its role in the auditory brainstem. This approach was motivated by the observation that the phospho-mimetic mutation of serine 937 is among the most effective ways to increase KCC2 activity in a heterologous expression system^65^, and since the auditory brainstem shows peculiarities concerning synaptic inhibition, such as posttranslational activation of KCC2 to induce D/H shift^47,49^ and the most negative E_Cl−_ observed so far in neurons^54^.

A fundamental process during development of the nervous system is the shift from depolarizing to hyperpolarizing action of inhibitory neurotransmitters. This is often accompanied by an increase in the expression or increase in the activity of KCC2^33,40–44,46^. In the auditory brainstem, especially in rat LSO neurons, the D/H shift occurs during the first postnatal week^8,20,74,75,84^. Here, KCC2 is perinatally expressed, but transport-inactive^47,49^. This raises the question how KCC2 activation is achieved during development^47,49^. Based on previous studies, three possible scenarios can be envisaged, which might be causally related: increase in the oligomer/monomer ratio^49^, a shift from membrane rafts to non-membrane rafts^50^, and phospho-regulation^51,85^.

Here, we hypothesized that the potent phospho-site serine 937 plays an important role and, therefore, generated KCC2 Thr^934Ala^/Ser^937Asp^ mice. These mice are viable and bred normally. They thus provide an excellent tool to explore in vivo the effect of phosphorylation on KCC2 activation and activity. By performing electrophysiological analyses in LSO neurons of acute brainstem slices, we showed that the developmental maturation of Cl^-^ dependent inhibition is shifted to earlier stages in homozygous KCC2 Thr^934Ala^/Ser^937Asp^ mice. These results are consistent with in vitro analyses, as the KCC2 Thr^934Ala^/Ser^937Asp^ double mutant leads to activation of KCC2 in HEK293 cells. Such enhanced KCC2 activity lowers the [Cl^−^]_i_ and increases the strength of glycinergic inhibition. Thus, phosphorylation of Ser^937^ is sufficient to induce KCC2 activity causing an earlier D/H shift in LSO neurons.

Similarly, (de)phosphorylation of Thr^906/^Thr^1007^ or Ser^940^ induce the onset of hyperpolarisation during development^86,87^. The decrease in phosphorylation of Thr^906^ and Thr^1007^^52,67^ increased KCC2 activity^60^. In transgenic KCC2 Thr^906Ala^/Thr^1007Ala^ mice, mimicking the dephosphorylated state of these two phospho-sites, an accelerated onset of hyperpolarisation in hippocampal neurons was observed, pointing to its importance for the D/H shift in this brain area^86^. In contrast, heterozygous KCC2 Thr^906Glu^/Thr^1007Glu^ mice, mimicking a phosphorylated state, showed a delayed D/H shift in CA3 pyramidal neurons and hippocampal slices^87^. These mice showed increased ultra-sonic vocalization, seizure susceptibility and long-term abnormalities in social behaviour^87^ in comparison to transgenic KCC2 Thr^906Ala/Thr1007Ala^ mice. In the latter, the dephosphorylation-mimicking status limits the onset and severity of seizure^64^. Ser^940^ is another phosphorylation site highly phosphorylated in cultured hippocampal neurons in the first postnatal week^86^. This enhances the KCC2 activity in cultured hippocampal neurons^69,86,88^. The importance of Ser^940^ phosphorylation is further corroborated by a delayed D/H shift in Ser^940Ala^ knock-in mice^86^. These mice suffer from profound social interaction abnormalities like in autism spectrum disorders^24,86^. Thus, phospho-regulation is a common mechanism to regulate KCC2 activity and to initiate the onset of hyperpolarisation. In view of its rich phospho-site tool box, it will be interesting to study in the future, whether individual phospho-sites have brain area-specific functions. The increasing number of site-specific KCC2 transgenic mice pave the way for such an analysis.

So far, it is unclear whether the stronger Cl^-^ extrusion in the transgenic KCC2^AD/AD^ mice has an implication for the auditory function. Presently, our gramicidin-perforated patch-clamp recordings revealed a conspicuous early D/H shift in KCC2^AD/AD^ mice (hyperpolarizing Cl^-^ gradient present at P3), suggesting early onset of KCC2-mediated Cl^-^ extrusion. This result implies a shorter depolarizing phase of inhibitory inputs onto LSO neurons during postmitotic differentiation. In this phase of neuronal maturation, GABA/glycine triggered depolarisation increase [Ca^2+^]_i_, thus contributing to synaptic maturation during development of neuronal circuits^9^. In the early postnatal LSO, GABA/glycine synaptic inputs evoke depolarizing EPSPs which induce Ca^2+^ transients spreading to dendritic processes and even trigger action potentials^8,15,20,89^. During the first postnatal week, such excitation likely facilitates NMDA-dependent developmental plasticity, thereby contributing to synaptic refinement of the MNTB-LSO projections^90^. It remains to be investigated whether a shorter depolarizing phase, caused by early KCC2 activity onset, affects the development of excitatory and inhibitory synapses on LSO or SPN neurons.

The D/H shift in the auditory brainstem is staggered in time, occurring before postnatal day 1 (P1) in the SPN to about P12 (i.e. at hearing onset) in the MNTB (cochlear nucleus:^48,76^; superior olivary complex:^8,47,74,91^). While the mechanisms underlying cell-type-specific regulation remain to be elucidated, these differences point to the importance of precise Cl^-^ homeostasis regulation for maturation of the brainstem circuit.

Another peculiarity of the auditory brainstem is an extremely negative E_Gly_ specifically in mature SPN neurons^54^. This is important for the precise offset firing after sound termination^53^. The neuron-type-specific regulation of E_Cl−_ in auditory brainstem neurons might be due to a different phosphorylation pattern of KCC2 that specifically attunes the strength of inhibition. Consistent with this notion, the phosphorylation status correlates with hyperpolarizing Cl^−^ gradient, as shown for E_GABA_ in adult hippocampal neurons of Thr^906Ala^/Thr^1007Ala^ transgenic mice^64^. Our analyses of KCC2^AD/AD^ mice demonstrate a more negative E_IPSC_ both in SPN and LSO neurons compared to KCC2^WT^. However, the present data do not explain the E_Cl−_ difference between SPN and LSO neurons, as the mutation caused a similar hyperpolarizing shift of about − 10 mV in both nuclei. Therefore, it is tempting to presume additional regulatory mechanisms rendering E_Gly_ more negative in the SPN. In the future, phospho-proteomic studies or phospho-site specific immunohistochemistry will be helpful to investigate the different phosphorylation pattern of KCC2 in SPN and LSO neurons. What effect will the more negative reversal potential have in SPN neurons? Remarkably, SPN neurons show very stable, strongly hyperpolarized IPSC reversal potentials after the D/H shift has taken place, without further changes after hearing onset around P12^92^. This suggests that the crucial window for adjusting the strength of inhibition is indeed around the D/H shift. Behavioural effects are to be expected in difficult sound localization tasks as well as gap-detection or sound duration discrimination tasks. Indeed, a reduction of KCC2 activity results in reduced gap detection in a cellular paradigm^54^. This loss of temporal resolution is present in ageing, hearing loss, and neurodegeneration^54^. Our results suggest that phosphorylation of Ser^937^ enhances KCC2 activity resulting in more negative reversal potential in SPN neurons, which in turn might enhance gap detection. Therefore, pharmaceuticals that mediate a neuronal-targeted increase in phosphorylation of Ser^937^ may represent a novel therapeutic strategy to prevent for example age-related hearing loss. In a high-throughput screening the compound CLP257 and its carbamate prodrug derivate CLP290 were found as promising KCC2 agonist compounds that enhance the activity of KCC2^93,94^ and increase the phosphorylation of Ser^940^^95^. However, a follow-up study did not confirm the effect of CLP257 on KCC2 activity^96^. Future studies should test whether this compound also has an influence on the phosphorylation of Ser^937^.

In conclusion, we showed that a phospho-mimetic mutation of serine 937 enhances KCC2 activity in the mammalian auditory brainstem. This shifted the onset of hyperpolarisation, i.e. E_Cl−_, to an earlier timepoint in development and rendered E_IPSC_ in mature LSO and SPN neurons more negative. Thus, phospho-regulation of KCC2 is a potent way to increase its transport activity. As such, pharmaceuticals that directly interfere with phosphorylation of KCC2 may be a novel therapeutic strategy.

## Material and methods

### Construction of the expression clones

Site-directed mutagenesis of mouse KCC2a (NM_001355480.1) was performed according to the QuikChange mutagenesis system (Stratagene, Heidelberg, Germany)^65,66,97^. The sequence for the forward oligonucleotide for the generation of the KCC2 Thr^934Ala^/Ser^937Asp^ mutant is CGGGAGATCCAGAGCATCGCAGACGAGGACCGGGGCTCCATTCGGAG. The generated clone was verified by sequencing (LGC genomics, Berlin).

### Cell culturing

For immunocytochemistry and measurement of K^+^-Cl^−^ cotransporter activity, HEK293 cells were transiently transfected with the respective constructs, using Turbofect (Fermentas, Schwerte, Germany). Cells were seeded in a 6-well plate 24 h prior transfection. The DMEM medium was replaced four hours before transfection. 150 µl Opti-MEM (Invitrogen, Karlsruhe, Germany), 6 µl Turbofect and the corresponding DNA amount were thoroughly mixed and incubated for 20 min at room temperature. The mixture was applied to the cells which were then shaken at 300 rpm for 10 min at room temperature.

For K^+^-Cl^−^ cotransporter activity measurements, transfected HEK293 cells were plated at a concentration of 1 × 10^5^ cells/well in a 0.1 mg/ml poly-L-lysine coated black-well 96 well culture dish (Greiner Bio-One, Frickenhausen, Germany) 24 h after transfection. Each transfected construct was plated out three times, and represents three technical replicates. For the generation of independent biological replicates, the constructs were transfected separately. At least five biological replicates were done. The remaining cells were plated on 0.1 mg/ml poly-L-lysine-coated glass coverslips. After ~ 18 h, coverslips were proceeded for immunocytochemical analysis to determine transfection rates, which were routinely between 20 and 30%.

### Immunocytochemistry

For immunocytochemistry, all steps were performed at room temperature. HEK293 cells grown on poly-L-lysine-coated coverslips were fixated for 10 min with 4% paraformaldehyde in 0.2 M phosphate buffer. Afterward, the cells were washed three times with PBS before the blocking solution (2% bovine serum albumin and 10% goat serum in PBS) was applied for 30 min. Primary antibody solution (anti-KCC2 N1-12; 1:1000; Neuromab, California, USA) was added in carrier solution (0.3% Triton X-100, 1% bovine serum albumin, 1% goat serum in PBS) and incubated for 1 h. After washing three times with PBS the secondary antibody, which was conjugated to a fluorescent probe (Alexa Flour 488 goat anti-mouse; 1:1000; Thermo Fisher Scientific, Bremen, Germany) was added to the carrier solution and incubated for 1 h. Again, the cells were washed three times with PBS and completely dried. The dried coverslips were mounted onto glass slides with Mowiol (Roth) and 4′,6-diamidine-2-phenylindole (DAPI, 1:1000; Roth). Photomicrographs were taken using an Olympus fluorescence microscope (Olympus BX63).

### Determination of the K^+^-Cl^-^cotransport activity

Transport activity of KCC2 was determined by Cl^−^-dependent uptake of Tl^+^ in HEK293 cells as described previously^72,73,98^. To initiate the flux measurement, the medium in the 96-well culture dish was replaced by 80 µl hypotonic preincubation buffer (100 mM N-methyl-D-glucamine-chloride, 5 mM (4-(2-hydroxyethyl)-1-piperazineethanesulfonic acid) (Hepes), 5 mM KCl, 2 mM CaCl_2_, 0.8 mM MgSO_4_, 5 mM glucose, pH 7.4; osmolarity: 175 mmol/kg ± 2) with 2 µM FluoZin-2 AM dye (Invitrogen) plus 0.2% (wt/vol) Pluronic F-127 (Invitrogen) and incubated for 48 min at RT. Afterward, cells were washed three times with 80 µl preincubation buffer and incubated for 15 min with 80 ml preincubation buffer including 0.1 mM ouabain to block the activity of the Na^+^/ K^+^ ATPase. Then, the 96-well plate was placed into a fluorometer (Fluoroskan FL, Thermo Scientific), and each well was injected with 40 µl 5 × Thallium stimulation buffer (12 mM Tl_2_SO_4_, 100 mM N-methyl-D-glucamine 5 mM Hepes, 2 mM KCl, 2 mM CaCl_2_, 0.8 mM MgSo_4_, 5 mM glucose, pH 7.4). The fluorescence was measured in a kinetic-dependent manner (excitation 485 nm, emission 538 nm, one frame in 6 s in a 200-speriod) across the entire cell population in a single well. By using linear regression of the initial values of the slope of Tl^+^- stimulated fluorescence increase, the transport activity was calculated.

*Statistical Analysis:* Transport activities of the respective mutant were tested against control samples (*mm*KCC2a^WT^ and mock), using a two-sample *t*-test after Student’s *t*-test for similar variances between samples. In exceptional cases, where the standard deviation differs by more than a factor of 2, Welch's *t*-test was used^99^. To avoid pseudo-replication, the number of degrees of freedom was deflated according to the size of independent preparations each with three technical replicates. The false-discovery rate was controlled and p-values were corrected using the Benjamini–Hochberg method^100^. The chance of false positive results (type 1 errors) was reduced by choosing *p*-values < 0.01.

The distribution of genotypes in the F1 generation was tested with the χ^2^ test under the null hypothesis of a Mendelian ratio of 1:2:1. The age-dependent weight (P0–P16) was tested with repeated measurement two-way ANOVA against the null hypothesis of no difference in mean weight, using sex, genotype, and the interaction of sex and genotype as fixed factors and the mouse id as random factor, i.e., *aov(weight* ~ sex*genotype + *Error(id))*.

### Animals

All protocols were in accordance with the German Animal Protection Law and approved by the local animal care and use committee (LAVES (Niedersächsisches Landesamt für Verbraucherschutz und Lebensmittelsicherheit), Oldenburg) and followed the NIH guide for the care and use of laboratory animals. Mice of both sexes were used. The KCC2^AD/AD^ mouse line was generated with CRISPR/Cas (clustered, regulatory interspaced, palindromic repeats (CRISPR)-associated (Cas) system) at the transgenic core facility at the Max Planck Institute of Molecular Cell Biology and Genetics (Dresden, Germany). For this purpose the guide RNA 5′cucguagugucugcucagag 3′ and the repair template: CATCCACTGTAGTAATGGCTCTTGGCAGGGCGTGGGTGGTGACCCCCCAGCAGAGCTGGCACCAACCTGTGTCACTCCCCAGATCCA**ATCG**AT**TG**C**C**GA**T**GA**AGAC**CG**A**GGCTCCATTCGGAGGAA was used. These mice were kept on a C57Bl/6N genetic background. Primers for genotyping KCC2 wild-type are as follows : for : ATGGGCCCTTGAAGGACAGG and rev : CCGAGACTCGTCTGTGATGCT. For the genotyping of the T934A/S937D mutation in *KCC2* the following primers were used: for: ATGGGCCCTTGAAGGACAGG, and rev: TCGGTCTTCATCGGCAATCGA.

### Morphometric volume analysis

Adult KCC2^AD/AD^ and KCC2^+/+^ mice (aged between postnatal day (P) 30–P60) were transcardially perfused with phosphate buffered saline (PBS; 136.9 mM NaCl, 2.7 mM KCl, 10.1 mM Na_2_HPO_4_, 1.8 mM KH_2_PO_4_, pH 7.4) followed by 4% paraformaldehyde solution. Brains were dissected and postfixed for 2–4 h in 4% PFA and stored in 30% sucrose in PBS for cryoprotection. The brainstem was cut in coronal sections with a thickness of 30 µm using a cryostat (Leica Biosystems, Wetzlar, Germany). Nissl staining was performed on consecutive sections of the auditory brainstem. Sections were imaged with an automated slide scanning microscope (AxioScan Z1, Carl Zeiss, Oberkochen, Germany). The volume of individual nuclei was obtained as described previously^101–103^. Shortly, images were analysed by outlining the auditory nuclei using Fiji^104^ and multiplying this area with the thickness of each section. Three animals per genotype were analysed. The experimentalist was blind to the respective genotype. Statistical analysis was performed using Student’s *t*-test after testing for Gaussian distribution using Prism version 9 (GraphPad, San Diego, California). Reported values are mean ± SD.

### Patch-clamp recordings

*Slice preparation:* Coronal slices (200 µm) containing the superior olivary complex including LSO and SPN were cut from P3 to P29 mice of either sex. The brainstem was sliced with a vibratome (Leica VT1200 S), in ice-cold, low-calcium artificial cerebrospinal fluid (ACSF) solution containing (in mM): 125 NaCl, 2.5 KCl, 0.1 CaCl_2_, 3 MgCl_2_, 1.25 NaH_2_PO_4_, 25 NaHCO_3_, 25 glucose, 2 sodium pyruvate, 3 myo-inositol, 0.5 ascorbic acid, continuously bubbled with 5% CO_2_ and 95% O_2_, pH 7.4. Slicing solution contained lower Ca^2+^ and higher Mg^2+^ concentration than the standard ACSF in order to avoid Ca^2+^-dependent signalling and activation of NMDAR. Slices were incubated in the standard recording solution (ACSF same as for slicing, except CaCl_2_ and MgCl_2_ were changed to 2 mM and 1 mM, respectively) for 30–45 min at 37 °C before being stored at room temperature until recordings.

*Gramicidin perforated patch recordings* were conducted at room temperature (22 ± 1 °C) in LSO neurons of P3 and P5 day old mice of both genotypes and either sex. Gramicidin perforated patch recordings were acquired as previously described^77,78^. In brief, patch pipettes were pulled with DMZ-Universal-Electrode horizontal puller from filamented borosilicate glass capillaries (Science Products) to have resistances of 5–6 MΩ when filled with a K^+^-gluconate-based internal solution. Pipettes were tip-filled as follows (mM): 97.5 potassium-gluconate, 32.5 KCl, 1 MgCl_2_, 10 HEPES, 5 EGTA, 10 HEPES, pH 7.34 with KOH (290 mOsm). The remainder of the pipette was back-filled with the same solution including gramicidin (50 µg/ml gramicidin A, Sigma) and 25 µM ATTO 488. The latter was used to confirm that the perforated patch was not ruptured. At the end of the experiment, the rupture of the perforated patch yielded E_Gly_ around the Nernst potential for Cl^−^ of − 34 mV, thus confirming that the gramicidin ionophores were impermeable for Cl^−^ and that our recordings were not affected by the [Cl^−^]_pip_. The progress of perforation was evaluated by monitoring the steady-state current responses to a − 5 mV voltage command (with access resistance dropping from 1 GΩ to around 20 MΩ). Recordings were done when series resistance reached a steady level (mean Rs = 20.8 ± 5 MΩ, n = 22), typically within 30–40 min after the giga-seal formation. Glycine (1 mM) was prepared in standard recording ACSF and pressure applied (100 ms) over the soma of recorded neuron using a Picospritzer (General Valve Corp.). Current responses to glycine puff were recorded while cells were held at different holding potentials (from − 82 to − 12 mV with increment of 10 mV, offline corrected for liquid junction potential). The constant stimulation conditions were assured by controlling the pipette diameter, application pressure and duration, and distance from the cell (3 µm, 5 psi, 5 ms, 10 µm, respectively). The perfusion was turned off just prior to each puff application to avoid unequal dilution of the agonist. Significance of the responses was determined by employing the *z*-test, i.e., level of acceptance was set at *z* <  − 3.3 (I_m_), which corresponds to *p* < 0.001 [*z* = (A-BL)/SD_BL_, with A being the maximal amplitude of the response, BL the mean of the baseline (2 s prior to stimulation), SD_BL_ the standard deviation of the baseline]. Pressure-ejection of ACSF under the same condition evoked no response (mean ACSF response =  − 0.34 ± 0.2 pA, *z* =  − 0.21 ± 0.13, *p* > 0.73, n = 4), while glycine evoked significant membrane currents in the same neurons.

*Synaptic stimulation experiments* were performed on LSO and SPN neurons of P14–P29 mice of both genotypes and either sex. Recordings were conducted at 37 ± 1 °C, maintained by an inline feedback temperature controller and heated stage (Warner Instruments) with the recording chamber being continuously perfused with ACSF at a rate of 1–2 ml min^−1^. Whole-cell patch-clamp recordings were made from visually identified LSO and SPN neurons. Patch pipettes were pulled from borosilicate glass capillaries (Warner Instruments) using a DMZ Universal electrode puller (Zeitz-Instuments Vertriebs GmbH), filled with an internal solution containing (in mM): K-gluconate 97.5, KCl 32.5, HEPES 40, EGTA 5, MgCl_2_ 1, Na_2_phosphocreatine 5, pH was adjusted to 7.2 with KOH. Synaptic responses were evoked by afferent fibre stimulation with concentric bipolar electrodes (FHC inc., #CBARC75). Voltage pulses were generated by the HEKA amplifier and post-amplified by an isolated pulse stimulator (AM Systems). Inhibitory currents were recorded in ACSF containing 6,7-dinitroquinoxaline-2,3-dione (DNQX; 10 µM) and D-2-amino-5-phosphonopentanoic acid (D-AP5; 50 µM) to block AMPA and NMDA glutamate receptors, respectively. Stated voltages are corrected for a liquid junction potential of − 11 mV.

The patch clamp recordings were acquired using a HEKA EPC 10 amplifier (HEKA Elektronik). Recorded signals were digitized at 50–100 kHz and filtered with a 6–10 kHz Bessel low-pass filter. Data were examined with FitMaster (HEKA) software followed by the analysis on the same software. Amplitude of the current responses from individual cells were plotted against holding potential under which they were recorded (IV diagram) and reversal potential was derived from linear fit of those values.

Statistics: Data sets were tested for Gaussian distribution prior to comparison with the Shapiro–Wilk test. Statistical analyses were carried out in Sigma Plot (Sigma Plot 14, Systat Software). Values are reported as mean ± SEM and compared using *t*-test (*p < 0.05, **p < 0.01, ***p < 0.001). The studies reported are in accordance with ARRIVE guidelines.

### Supplementary Information

Supplementary Information 1.

Supplementary Information 2.

Supplementary Information 3.

## Data Availability

All data generated or analysed during this study are included in this published article and its supplementary information files.
